# Diagnostic Value of Methylated Human Telomerase Reverse Transcriptase in Human Cancers: A Meta-Analysis

**DOI:** 10.3389/fonc.2015.00296

**Published:** 2015-12-24

**Authors:** Wei Gao, Yuan Shi, Wei Liu, Wei-Yin Lin, Josh Chia-Ching Wu, Jimmy Yu-Wai Chan, Thian-Sze Wong

**Affiliations:** ^1^Department of Surgery, The University of Hong Kong, Hong Kong, China; ^2^Department of Plastic and Reconstructive Surgery, Shanghai Jiaotong University School of Medicine, Shanghai, China; ^3^Department of Cell Biology and Anatomy, National Cheng-Kung University, Taiwan, China

**Keywords:** hTERT, promoter methylation, diagnostic accuracy, meta-analysis, qMSP assay

## Abstract

Human telomerase reverse transcriptase (hTERT) plays a critical role in the pathogenesis of human malignancies. Overexpression of hTERT is essential in controlling the propagation of cancer cells. The CpG island located at hTERT promoter region is subjected to methylation modification in human cancer. In this perspective article, we discussed the diagnostic value of methylated hTERT in human cancers. The definitive diagnosis of most solid tumors is based on histological and immunohistochemical features. Under certain circumstances, however, the use of methylated hTERT might be useful in overcoming the limitation of the conventional methods. Methylated hTERT showed a good diagnostic power in discriminating cancer from benign or normal tissues. Nevertheless, differences in detection method, methylation site, cancer type, and histological subtype of cancer make it difficult to evaluate the actual diagnostic accuracy of methylated hTERT. Therefore, we performed subgroup analysis to assess the effects of these factors on the diagnostic efficiency of methylated hTERT. We demonstrated that quantitative MSP (qMSP) assay offers the highest discriminative power between normal and cancer in comparison with different detection methods. In addition, the methylated sites selected by different studies had an impact on the detection performance. Moreover, the diagnostic power of methylated hTERT was affected by cancer type and histological subtype. In conclusion, the existing evidence demonstrated that methylated hTERT is effective in cancer detection. Detailed profiling of the methylation sites to local the common methylation hotspot across human cancers is warranted to maximize the diagnostic value of methylated hTERT in cancer detection.

## Introduction

Cancer is a leading cause of death, accounting for about 14.6% of all human deaths (1). Effective medical intervention could only be achieved if the cancerous tissue is identified in advance. Molecular screening with biomarkers is now recognized as an efficient means in early cancer detection. Biomarkers of which expression changes have close linkage with the progression of cancer phenotype are adopted as an indicator of cancers.

Human telomerase reverse transcriptase (hTERT) activation is one of the universal and hallmark changes in cancer progression. Telomerase is the enzyme responsible for maintaining chromosomal endings during cell replication. Telomerase activation could lead to uncontrolled proliferation and immortalization through inhibition of replicative senescence (2, 3). Most mature normal cells silence the telomerase and their telomerase expression remains low (4). The telomerase activity is controlled directly by the transcript quantity of hTERT gene. Hence, increased hTERT transcript level is an indication of cancer cells and reflects the proliferative propensity accordingly. Clinically, high hTERT level in the cancerous tissue is associated with the poor outcome in a number of human cancers (5–8).

The gene encoding hTERT is regulated by multiple mechanisms. Activation of hTERT transcription requires the cooperation between multiple regulatory proteins. The promoter region of hTERT gene is CpG rich and harbors distinct CpG islands. The cytosine of CpG dinucleotides in the hTERT CpG islands is susceptible to methylation modification. Methylated CpG islands are usually observed in silenced genes as the aberrant addition of methyl group has a negative impact on transcription initiation. Furthermore, the methylated sequence allows the binding of specific transcription suppressors (9, 10). By contrast, methylated hTERT promoter has a positive impact on its activation because methylation prevents the binding of CTCF repressor (11). The methylated promoter DNA allows the binding of specific activators and regulatory proteins (e.g., c-Myc, Sp1) that regulate hTERT transcription directly (12). This feature is cancer specific and is absent in most of the hTERT gene in the normal somatic cells (13, 14). Hence, the presence of methylated hTERT DNA is a potential indicator for the presence of cancer cells.

In comparison with other forms of cancer biomarker, methylated gene is advantageous for the high cancer specificity. Aberrant DNA methylation is a covalent modification. The diagnostic performance of methylated DNA however varies in a gene-by-gene and cancer-specific manner. Not all the methylated CpG sites/island can lead to the expression activation of hTERT gene. For example, methylation of the E-box in hTERT promoter will lead to a weak transcription activation as the interaction between C-Myc and E-box was reduced due to the presence of methylated moiety (12). Thus, selection between different hTERT methylation sites will affect the resulting diagnostic accuracy. At present, the use of hTERT DNA as cancer biomarker remains controversial as the diagnostic performance varies depending on the methylated loci (13, 15). In addition, studies across different cancer types examining different hTERT hotspot make it difficult to comprehend and evaluate the actual diagnostic accuracy of hTERT for cancer detection.

In the current study, we first performed a systematic review on the methylated hTERT hotspots in human malignancies. Furthermore, meta-analysis was used to evaluate the diagnostic performance of hTERT in cancer detection.

## Methylated hTERT Showed a Good Diagnostic Power in Discriminating Cancer from Benign OR Normal Tissues

Systematic search from PubMed was performed using the key terms included “cancer,” “carcinoma,” “tumor,” “neoplasm,” “methylation,” “hypermethylation,” “hypomethylation,” “demethylation,” “TERT,” and “telomerase reverse transcriptase.” In total, 290 articles were identified from PubMed search (Figure S1 in Supplementary Material). According to our selection criteria, we excluded 278 records due to language (*n* = 9), no full-texts (*n* = 45), review article (*n* = 35), and irrelevant articles (*n* = 189). Subsequently, the remaining 12 relevant studies with full text were assessed. The pooled sensitivity (Se), specificity (Sp), and diagnostic odds ratio (DOR) were calculated using MetaDiSc software (Version 1.4) (16). Oikonomou et al. (17) were excluded due to the fact that the study population is duplicated by Iliopoulos et al. (18). Only studies focused on hTERT core regulatory promoter (−1876 to +335) relative to translation start site ATG were included (19). Studies by Iliopoulos et al. were excluded as the examined hTERT methylation site was out of our scope (18). Finally, our systematic search yielded 10 studies (20–29). Table S1 in Supplementary Material displayed the detailed characteristics of each study. The primers and probes used for quantitative MSP (qMSP) assay in different studies were listed in Table S3 in Supplementary Materials. The studies quality was evaluated using the revised Quality Assessment for Studies of Diagnostic Accuracy tool (QUADAS-2), and the results were listed in Table S2 in Supplementary Material (30).

**Figure 1:**
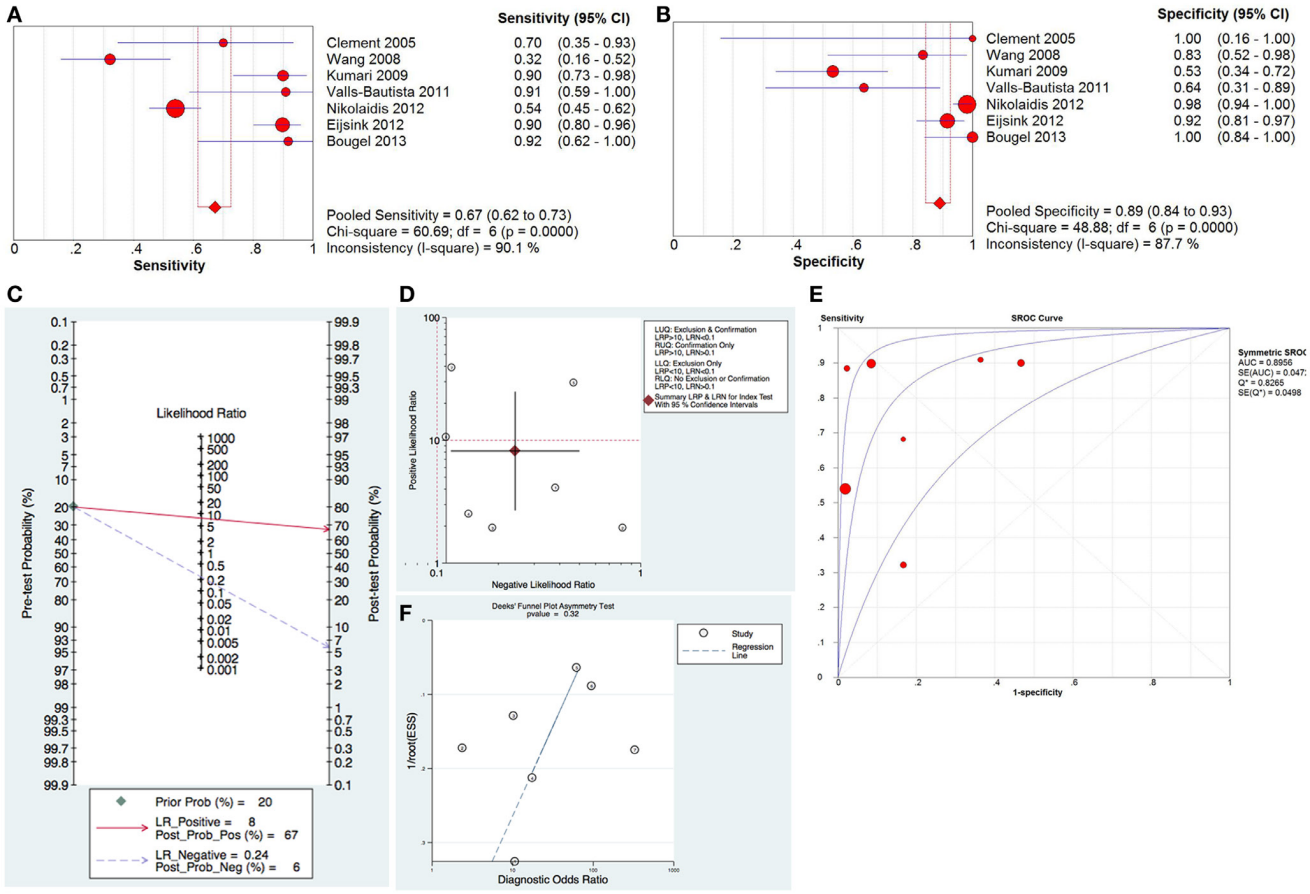
**Diagnostic value of methylated hTERT for distinguishing cancer from normal**. Forest plot of sensitivity **(A)** and specificity **(B)** of methylated hTERT for discriminating cancer from normal. **(C)** Fagan plot displaying post-test probability. **(D)** Likelihood ratio scattergram for confirmation and exclusion. **(E)** SROC curve for diagnostic accuracy. **(F)** Funnel plot with superimposed regression line for testing publication bias.

For the diagnostic value of methylated hTERT in distinguishing cancer from normal, 7 studies with 543 samples were included in this meta-analysis. The pooled sensitivity and specificity of methylated hTERT were 0.67 (95%CI, 0.62–0.73) and 0.89 (95% CI, 0.84–0.93), respectively (Figures 1A,B). The DOR was 24.71 (95% CI, 7.39–82.63). STATA (Version 12, STATA Crop., USA) was used to generate the Fagan plot and likelihood ratio scattergram. The pooled positive likelihood ratio (PLR) and negative likelihood ratio (NLR) were 8 and 0.24, respectively (Figure 1C). As indicated by the pooled PLR value of the seven studies, patients with cancer have an eightfold higher chance of being methylated hTERT positive in comparison with non-cancer controls. The pooled NLR value of 0.24 indicates that the probability of having cancer is 24% when methylated hTERT was negative. The summary likelihood matrix point was located in the right lower quadrant (PLR < 10 and NLR > 0.1) (Figure 1D), indicating that methylated hTERT is neither useful for confirming the presence of cancer nor useful as an exclusion indicator. In addition, the summary receiver operation characteristic (SROC) curve was generated based on sensitivity and specificity of each study. The area under the curve (AUC) of SROC curve was 0.90 (SE = 0.05) (Figure 1E). Publication bias was examined by Deek’s funnel plot asymmetry test. No publication bias was observed among the selected studies (*p* = 0.32) (Figure 1F). Heterogeneity was assessed by *I*^2^ statistic. If *I*^2^ value was lower than 50%, the study was considered as homogeneous and Mantel–Haenszel model was applied. If *I*^2^ value was higher than 50%, the study was regarded as high heterogeneity and the Dersimonian–Laird model was adopted instead. Heterogeneity was observed in both sensitivity and specificity of methylated hTERT (Figures 1A,B). Threshold evaluation was used to examine the heterogeneity in diagnostic tests (16). Threshold effect was evaluated by calculating Spearman correlation coefficient between logit of sensitivity and logit of 1-specificity. The Spearman correlation coefficient of logit of sensitivity and logit of 1-specificity of methylated hTERT was 0.54 (*p* = 0.21), indicating that there is no heterogeneity from threshold effect.

For the diagnostic performance of methylated hTERT in discriminating cancer from benign tissue, the pooled sensitivity and specificity were 0.57 (95% CI, 0.49–0.65) and 0.81 (95% CI, 0.75–0.86), respectively (Figures S2A,B in Supplementary Material). The DOR was 19.54 (95% CI, 9.53–40.07). The pooled PLR and NLR were 10 and 0.59, respectively (Figure S2C in Supplementary Material). The summary likelihood matrix point was located in the right upper quadrant (PLR > 10 and NLR > 0.1) (Figure S2D in Supplementary Material). The AUC of SROC curve was 0.88 (SE = 0.03) (Figure S2E in Supplementary Material). A *p* value of 0.14 from Deek’s funnel plot asymmetry test suggested no publication bias among studies (Figure S2F in Supplementary Material). Heterogeneity was noticed in sensitivity and specificity (Figures S2A,B in Supplementary Material). Threshold effect was not a source of heterogeneity (Spearman correlation coefficient = 0.60, *p* = 0.40).

Methylated hTERT has a good diagnostic power in discriminating cancer from normal tissues (DOR: 24.71 and AUC: 0.9). The DOR is the ratio of the odds of a true-positive to the odds of a false-positive. The value of DOR ranges from 0 to infinity. Higher value indicates better discriminatory performance. The AUC from the ROC curve is an indicator of diagnostic accuracy (AUC > 0.97: excellent accuracy, 0.93–0.96: very good, 0.75–0.92: good) (31). The diagnostic accuracy, however, is weaker for discriminating cancer tissues from benign tissues (DOR: 19.54 and AUC: 0.88). The diagnostic power is lowest to differentiate benign and normal tissues (DOR: 3.24 and AUC: 0.57). The definitive diagnosis of most solid tumor is based on histological and immunohistochemical features. Under certain circumstances, however, the use of molecular markers might be useful in overcoming the limitation of the conventional methods. For example, fine needle aspirate cytology (FNAC) is a major methods used in salivary gland tumor diagnosis. It is difficult, however, to distinguish patients with carcinoma ex pleomorphic adenoma (Ca ex PSA) from benign pleomorphic salivary adenomas (PSA) through cytological examination on the aspirate (27). Although FNAC has a high diagnostic accuracy for histologically high-grade Ca ex PSA, its diagnostic efficiency decreases in low-grade Ca ex PSA. Methylated TERT is only present in the Ca ex PSA and is undetectable in benign PSA. Thus, the use of methylated TERT as adjuvant marker may provide clinical subtype information for personalized management of this disease (27).

## Effects of Detection Methods on the Diagnostic Efficiency of Methylated hTERT

Different methods were employed to evaluate the methylation status of the hTERT promoter. Six studies used qMSP assay (20, 22, 23, 25–27). Other detection methods, including methylation-sensitive dot blot assay (MS-DBA), 3D microarray, methylation-sensitive single-strand conformation analysis (MS-SSCA), and MS-PCR, were employed by the remaining four studies.

For qMSP assay, the pooled sensitivity and specificity were 0.67 (95% CI, 0.61–0.73) and 0.96 (95% CI, 0.93–0.98), respectively, for distinguishing cancer from normal tissues (Figures S3A,B in Supplementary Material). The DOR from studies using qMSP assay was 80.86 (95% CI, 31.01–210.86) (Figure S3C in Supplementary Material). The value is remarkably higher than studies using non-qMSP assays (range: 2.37–17.5) as detection mean (Table 1). AUC from studies using qMSP assay (0.97) was obviously higher in comparison with the non-qMSP studies (range: 0.58–0.85) (Table 1).

**Table 1:** **Diagnostic value of methylated hTERT for cancer, benign and normal**.

Group	No. of studies	Sample size	Sensitivity (95% CI)	Specificity (95% CI)	Diagnostic OR (95% CI)	AUC (SE)
**Overall**						
Cancer vs. normal	7	543	0.67 (0.62−0.73)	0.89 (0.84–0.93)	24.71 (7.39–82.63)	0.90 (0.05)
Cancer vs. benign	4	356	0.57 (0.49–0.65)	0.81 (0.75–0.86)	19.54 (9.53–40.07)	0.88 (0.03)
Benign vs. normal	1	202	0.23 (0.16–0.31)	0.92 (0.81–0.97)	3.24 (1.20–8.77)	0.57 (0.04)
**Detection methods**						
*qMSP*						
Cancer vs. normal	3	409	0.67 (0.61–0.73)	0.96 (0.93–0.98)	80.86 (31.01–210.86)	0.97 (0.02)
*MS-DBA*						
Cancer vs. normal	1	12	0.70 (0.35–0.93)	1.00 (0.16–1.00)	10.71 (0.40–287.83)	0.85 (0.12)
*MS-PCR*						
Cancer vs. normal	1	60	0.90 (0.73–0.98)	0.53 (0.34–0.72)	10.29 (2.56–41.37)	0.72 (0.07)
*MS-SSCA*						
Cancer vs. normal	1	22	0.91 (0.59–1.00)	0.64 (0.31–0.89)	17.50 (1.60–191.89)	0.77 (0.11)
*3D microarray*						
Cancer vs. normal	1	40	0.32 (0.16–0.52)	0.83 (0.52–0.98)	2.37 (0.43–13.13)	0.58 (0.10)
**Methylation sites (amplicon location)**						
(**−*383/*−*295*)						
Cancer vs. normal	1	128	0.90 (0.80–0.96)	0.92 (0.81–0.97)	95.66 (28.69–318.94)	0.91 (0.03)
Cancer vs. benign	2	255	0.89 (0.80–0.94)	0.76 (0.69–0.82)	24.40 (11.60–51.31)	*NA*
(**−*380/*−*280*)						
Cancer vs. normal	1	248	0.54 (0.45–0.62)	0.98 (0.94–1.00)	62.70 (14.88–264.09)	0.76 (0.03)
Cancer vs. benign	1	59	0.13 (0.04–0.30)	1.00 (0.88–1.00)	9.33 (0.48–181.51)	0.57 (0.08)
(**−*540/*−*440*)						
Cancer vs. normal	1	33	0.92 (0.62–1.00)	1.00 (0.84–1.00)	329.67 (12.41–8758.83)	0.96 (0.05)
(**−*346/*−*273*)						
Cancer vs. benign	1	42	0.04 (0.00–0.19)	1.00 (0.78–1.00)	1.75 (0.07–45.77)	0.52 (0.09)
**Cancer type**						
Cervical cancer						
Cancer vs. normal	1	128	0.90 (0.80–0.96)	0.92 (0.81–0.97)	95.66 (28.69–318.94)	0.91 (0.03)
Cancer vs. benign	2	255	0.89 (0.80–0.94)	0.76 (0.69–0.82)	24.40 (11.60–51.31)	*NA*
Lung cancer						
Cancer vs. normal	2	288	0.50 (0.42–0.58)	0.97 (0.92–0.99)	12.59 (0.47–340.18)	*NA*
Leptomeningeal metastases						
Cancer vs. normal	1	33	0.92 (0.62–1.00)	1.00 (0.84–1.00)	329.67 (12.41–8758.83)	0.96 (0.05)
Colorectal cancer						
Cancer vs. Normal	1	22	0.91 (0.59–1.00)	0.64 (0.31–0.89)	17.50 (1.60–191.89)	0.77 (0.11)
Salivary glands carcinoma						
Cancer vs. benign	1	59	0.13 (0.04–0.30)	1.00 (0.88–1.00)	9.33 (0.48–181.51)	0.57 (0.08)
Pancreatic cancer						
Cancer vs. normal	1	60	0.90 (0.73–0.98)	0.53 (0.34–0.72)	10.29 (2.56–41.37)	0.72 (0.07)
Mesothelioma						
Cancer vs. benign	1	42	0.04 (0.00–0.19)	1.00 (0.78–1.00)	1.75 (0.07–45.77)	0.52 (0.09)
*Esophageal adenocarcinoma*						
Cancer vs. normal	1	12	0.70 (0.35–0.93)	1.00 (0.16–1.00)	10.71 (0.40–287.83)	0.85 (0.12)
**Histological subtype of lung cancer**						
Adenocarcinoma						
Cancer vs. normal	1	27	0.33 (0.12–0.62)	0.83 (0.52–0.98)	2.50 (0.39–16.05)	0.58 (0.11)
Squamous cell carcinoma						
Cancer vs. normal	1	19	0.43 (0.10–0.82)	0.83 (0.52–0.98)	3.75 (0.44–31.62)	0.63 (0.14)

*qMSP, quantitative methylation-specific PCR; MS-SSCA, methylation-sensitive single-strand conformation analysis; MS-PCR, methylation-specific PCR; MS-DBA, methylation-sensitive dot blot assay*.

These results indicated that different detection methods affected the diagnostic efficiency of methylated hTERT. Among all the reported methods used in detecting methylated hTERT, qMSP assay demonstrated the highest diagnostic accuracy in comparison with other detection methods (as evidenced by the DOR and AUC values). As the studies employing non-qMSP methods (such as MS-DBA and MS-SSCA) only contain a few cases and the number of similar studies remains very limiting, further comparative studies at a larger scale are essential to affirm the performance of qMSP. An AUC value of 0.97 revealed that the diagnostic accuracy of methylated hTERT examination using qMSP assay is excellent in discriminating cancer from normal tissues (31).

## Differential Diagnostic Power of Different qMSP Primer Probe Sets

Among the six studies using qMSP to detect methylated hTERT, different primer probe sets were adopted (Table S3 and Figure S4 in Supplementary Material). For discrimination between cancer and normal, primers and probe set flanking −540/−440 yielded the highest diagnostic accuracy as evidenced by the highest value of DOR (329.67) and AUC (0.96); for distinguishing cancer from benign tissue, primer probe set flanking −383/−295 exhibited the highest diagnostic efficiency with highest value of DOR (24.40) (Table 1).

Different primer probe sets used in the qMSP assays affect the diagnostic performance of methylated hTERT. This could possibly be because of the different consequent events associated with the methylation of distinct sites of the hTERT promoter. Primers with amplicon located at −540/−440 and −383/−295 displayed a higher diagnostic accuracy. The promoter region −540/−440 contains activator protein 2 (AP2) and nuclear factor 1 (NF1)-binding sites, while promoter region −383/−295 harbors Ikaros 2 (IK2), AP2 and activator protein 4 (AP4)-binding sites (32). The promoter −380/−280 also contained IK2-binding sites. AP2, NF1, and AP4 are transcription factors that could activate hTERT transcription (33). It has been reported that methylation at AP2-binding sites suppressed the binding of AP-2 (34). These results indicated that methylation at specific binding site could alter the binding with its transcription factors, leading to positive regulation of hTERT gene expression. Nevertheless, the regulatory elements that are essential for the regulation of hTERT gene and the diagnostic efficacy of methylated hTERT need to be delineated by functional experiments such as *in vitro* methylation assay.

## The Diagnostic Performance of Methylated hTERT in Various Cancers

Two studies investigated the diagnostic value of methylated hTERT in both cervical cancer and lung cancer. Only one study was available for each of the other cancer types. High variations in DOR values (ranged from 1.75 to 329.67) and the AUC values (ranged from 0.52 to 0.96) were observed between different cancer types (Table 1). Given that the diagnostic accuracy of methylated TERT might vary in the histological subtype of each cancer type, we intended to stratify each cancer type depending on the histological or molecular subtypes and evaluate the performance difference of methylated hTERT. Among the 10 included studies, only Wang et al. (27) provided histological information for subsequent subgroup analysis. Accordingly, their lung cancer cohorts could be stratified into two groups: adenocarcinoma and squamous cell carcinoma of which, methylated hTERT had a remarkably higher value in sensitivity, DOR, and AUC in lung squamous cell carcinoma in comparison with adenocarcinoma (Table 1).

## Conclusion

Our meta-analysis reveals that methylated hTERT displays diagnostic efficacy in cancer detection and qMSP assay exhibits the highest discriminative power between normal and cancer tissues. Nevertheless, one major limitation of the current study is that the sample size of single study is small. The performance of methylated hTERT as a diagnostic biomarker is highly varying relying on the correct selection of methylation hotspots. In order to use methylated hTERT as a universal diagnostic or screening marker, detail methylation profiling is warranted to define the common hTERT methylation hotspots in order to maximize the performance of the methylated hTERT as a biomarker in cancer detection.

## Author Contributions

T-SW conceived the study. WG, YS, and W-YL reviewed and extracted data from the literature. WG, YS, WL, W-YL, and JW carried out the meta-analysis and interpretation of the data. T-SW, WG, W-YL, and JC drafted and revised the manuscript. All authors read and approved the final manuscript.

## Conflict of Interest Statement

The authors declare that the research was conducted in the absence of any commercial or financial relationships that could be construed as a potential conflict of interest. The reviewer Alessandro Rimessi and handling Editor Paolo Pinton declared their shared affiliation, and the handling Editor states that the process nevertheless met the standards of a fair and objective review.

## References

[1] Stewart BW Wild CP World Cancer Report 2014. Lyon: World Health Organization; IARC Nonserial Publication (2014).

[2] Hahn WC Stewart SA Brooks MW York SG Eaton E Kurachi A Inhibition of telomerase limits the growth of human cancer cells. Nat Med (1999) 5(10):1164–70. 10.1038/13495 10502820

[3] Harley CB Kim NW Prowse KR Weinrich SL Hirsch KS West MD Telomerase, cell immortality, and cancer. Cold Spring Harb Symp Quant Biol (1994) 59:307–15. 10.1101/SQB.1994.059.01.035 7587082

[4] Harley CB Telomerase and cancer therapeutics. Nat Rev Cancer (2008) 8(3):167–79. 10.1038/nrc2275 18256617

[5] Ducrest AL Szutorisz H Lingner J Nabholz M. Regulation of the human telomerase reverse transcriptase gene. Oncogene (2002) 21(4):541–52. 10.1038/sj.onc.1205081 11850779

[6] Gertler R Rosenberg R Stricker D Friederichs J Hoos A Werner M Telomere length and human telomerase reverse transcriptase expression as markers for progression and prognosis of colorectal carcinoma. J Clin Oncol (2004) 22(10):1807–14. 10.1200/JCO.2004.09.160 15143073

[7] Sanders RP Drissi R Billups CA Daw NC Valentine MB Dome JS. Telomerase expression predicts unfavorable outcome in osteosarcoma. J Clin Oncol (2004) 22(18):3790–7. 10.1200/JCO.2004.03.043 15365076

[8] Tabori U Ma J Carter M Zielenska M Rutka J Bouffet E Human telomere reverse transcriptase expression predicts progression and survival in pediatric intracranial ependymoma. J Clin Oncol (2006) 24(10):1522–8. 10.1200/JCO.2005.04.2127 16575002

[9] Jones PA Baylin SB. The fundamental role of epigenetic events in cancer. Nat Rev Genet (2002) 3(6):415–28. 10.1038/nrg816 12042769

[10] Wajed SA Laird PW DeMeester TR. DNA methylation: an alternative pathway to cancer. Ann Surg (2001) 234(1):10–20. 10.1097/00000658-200107000-00003 11420478 PMC1421942

[11] Renaud S Loukinov D Abdullaev Z Guilleret I Bosman FT Lobanenkov V Dual role of DNA methylation inside and outside of CTCF-binding regions in the transcriptional regulation of the telomerase hTERT gene. Nucleic Acids Res (2007) 35(4):1245–56. 10.1093/nar/gkl1125 17267411 PMC1851636

[12] Wang Z Xu J Geng X Zhang W. Analysis of DNA methylation status of the promoter of human telomerase reverse transcriptase in gastric carcinogenesis. Arch Med Res (2010) 41(1):1–6. 10.1016/j.arcmed.2009.11.001 20430247

[13] Dessain SK Yu H Reddel RR Beijersbergen RL Weinberg RA. Methylation of the human telomerase gene CpG island. Cancer Res (2000) 60(3):537–41. 10676632

[14] Devereux TR Horikawa I Anna CH Annab LA Afshari CA Barrett JC. DNA methylation analysis of the promoter region of the human telomerase reverse transcriptase (hTERT) gene. Cancer Res (1999) 59(24):6087–90. 10626795

[15] Guilleret I Yan P Grange F Braunschweig R Bosman FT Benhattar J. Hypermethylation of the human telomerase catalytic subunit (hTERT) gene correlates with telomerase activity. Int J Cancer (2002) 101(4):335–41. 10.1002/ijc.10593 12209957

[16] Zamora J Abraira V Muriel A Khan K Coomarasamy A. Meta-DiSc: a software for meta-analysis of test accuracy data. BMC Med Res Methodol (2006) 6:31. 10.1186/1471-2288-6-31 16836745 PMC1552081

[17] Oikonomou P Messinis I Tsezou A DNA methylation is not likely to be responsible for hTERT expression in premalignant cervical lesions. Exp Biol Med (Maywood) (2007) 232(7):881–6. 17609503

[18] Iliopoulos D Oikonomou P Messinis I Tsezou A Correlation of promoter hypermethylation in hTERT, DAPK and MGMT genes with cervical oncogenesis progression. Oncol Rep (2009) 22(1):199–204. 10.3892/or_00000425 19513524

[19] Guilleret I Benhattar J. Unusual distribution of DNA methylation within the hTERT CpG island in tissues and cell lines. Biochem Biophys Res Commun (2004) 325(3):1037–43. 10.1016/j.bbrc.2004.10.137 15541393

[20] Bougel S Lhermitte B Gallagher G de Flaugergues JC Janzer RC Benhattar J. Methylation of the hTERT promoter: a novel cancer biomarker for leptomeningeal metastasis detection in cerebrospinal fluids. Clin Cancer Res (2013) 19(8):2216–23. 10.1158/1078-0432.CCR-12-1246 23444211

[21] Clement G Benhattar J. A methylation sensitive dot blot assay (MS-DBA) for the quantitative analysis of DNA methylation in clinical samples. J Clin Pathol (2005) 58(2):155–8. 10.1136/jcp.2004.021147 15677535 PMC1770569

[22] Eijsink JJ Lendvai A Deregowski V Klip HG Verpooten G Dehaspe L A four-gene methylation marker panel as triage test in high-risk human papillomavirus positive patients. Int J Cancer (2012) 130(8):1861–9. 10.1002/ijc.26326 21796628

[23] Eijsink JJ Yang N Lendvai A Klip HG Volders HH Buikema HJ Detection of cervical neoplasia by DNA methylation analysis in cervico-vaginal lavages, a feasibility study. Gynecol Oncol (2011) 120(2):280–3. 10.1016/j.ygyno.2010.10.029 21093897

[24] Kumari A Srinivasan R Vasishta RK Wig JD. Positive regulation of human telomerase reverse transcriptase gene expression and telomerase activity by DNA methylation in pancreatic cancer. Ann Surg Oncol (2009) 16(4):1051–9. 10.1245/s10434-009-0333-8 19194757

[25] Nikolaidis G Raji OY Markopoulou S Gosney JR Bryan J Warburton C DNA methylation biomarkers offer improved diagnostic efficiency in lung cancer. Cancer Res (2012) 72(22):5692–701. 10.1158/0008-5472.CAN-12-2309 22962272 PMC3500566

[26] Pu RT Sheng ZM Michael CW Rhode MG Clark DP O’Leary TJ. Methylation profiling of mesothelioma using real-time methylation-specific PCR: a pilot study. Diagn Cytopathol (2007) 35(8):498–502. 10.1002/dc.20692 17636483

[27] Schache AG Hall G Woolgar JA Nikolaidis G Triantafyllou A Lowe D Quantitative promoter methylation differentiates carcinoma ex pleomorphic adenoma from pleomorphic salivary adenoma. Br J Cancer (2010) 103(12):1846–51. 10.1038/sj.bjc.6605953 21063414 PMC3008600

[28] Valls-Bautista C Bougel S Pinol-Felis C Vinas-Salas J Benhattar J. hTERT methylation is necessary but not sufficient for telomerase activity in colorectal cells. Oncol Lett (2011) 2(6):1257–60. 10.3892/ol.2011.386 22848298 PMC3406488

[29] Wang Y Zhang D Zheng W Luo J Bai Y Lu Z. Multiple gene methylation of nonsmall cell lung cancers evaluated with 3-dimensional microarray. Cancer (2008) 112(6):1325–36. 10.1002/cncr.23312 18286531

[30] Whiting PF Rutjes AW Westwood ME Mallett S Deeks JJ Reitsma JB QUADAS-2: a revised tool for the quality assessment of diagnostic accuracy studies. Ann Intern Med (2011) 155(8):529–36. 10.7326/0003-4819-155-8-201110180-00009 22007046

[31] Jones CM Athanasiou T. Summary receiver operating characteristic curve analysis techniques in the evaluation of diagnostic tests. Ann Thorac Surg (2005) 79(1):16–20. 10.1016/j.athoracsur.2004.09.040 15620907

[32] Cong YS Wen J Bacchetti S. The human telomerase catalytic subunit hTERT: organization of the gene and characterization of the promoter. Hum Mol Genet (1999) 8(1):137–42. 10.1093/hmg/8.1.137 9887342

[33] Poole JC Andrews LG Tollefsbol TO. Activity, function, and gene regulation of the catalytic subunit of telomerase (hTERT). Gene (2001) 269(1–2):1–12. 10.1016/S0378-1119(01)00440-1 11376932

[34] Comb M Goodman HM. CpG methylation inhibits proenkephalin gene expression and binding of the transcription factor AP-2. Nucleic Acids Res (1990) 18(13):3975–82. 10.1093/nar/18.13.3975 1695733 PMC331101

