# Supplementary material for: Diagnostic Value of Methylated Human Telomerase Reverse Transcriptase in Human Cancers: A Meta-Analysis
Source: Front Oncol. 2015 Dec 24;5:296. doi: 10.3389/fonc.2015.00296 (PMC4689846; doi:10.3389/fonc.2015.00296)
Supplement: Supplementary file 1 [file table_1.docx]

**Table S1. Characteristics of studies included in this study.**

| First author | Year of study | Country | Patient age (y) | Detection method | Tumor | Control | Sample type | Cancer type | Reference |
| --- | --- | --- | --- | --- | --- | --- | --- | --- | --- |
| Bougel | 2013 | Switzerland | unclear | qMSP | 11 | 21 | Cerebrospinal fluids | Leptomeningeal metastases | 20 |
| Nikolaidis | 2012 | United Kingdom | 68.4±8.07 | qMSP | 139 | 109 | Tissue | Lung cancer | 25 |
| Eijsink | 2012 | Netherlands | 47(27-85) | qMSP | 74 | 217 | Tissue | Cervical cancer | 22 |
| Eijsink | 2011 | Netherlands | 45(22-85) | qMSP | 20 | 23 | Cervical cancer lavages | Cervical cancer | 23 |
| Valls-Bautista | 2011 | Spain | 75.4 (65-82) | MS-SSCA | 11 | 11 | Tissue | Colorectal cancer | 28 |
| Schache | 2010 | United Kingdom | unclear | qMSP | 31 | 28 | Tissue | Salivary glands carcinoma | 27 |
| Kumari | 2009 | India | unclear | MS-PCR | 30 | 30 | Tissue | Pancreatic cancer | 24 |
| Wang | 2008 | China | unclear | 3D microarray | 28 | 12 | Tissue | Lung cancer | 29 |
| Pu | 2007 | USA | unclear | qMSP | 27 | 15 | Tissue | Mesothelioma | 26 |
| Clement | 2005 | Switzerland | unclear | MS-DBA | 10 | 2 | Tissue | Oesophageal adenocarcinoma | 21 |

qMSP: Quantitative methylation specific PCR; MS-SSCA: Methylation-sensitive single-strand conformation analysis; MS-PCR: Methylation-specific PCR; MS-DBA: Methylation sensitive dot blot assay.
