# Supplementary material for: Diagnostic Value of Methylated Human Telomerase Reverse Transcriptase in Human Cancers: A Meta-Analysis
Source: Front Oncol. 2015 Dec 24;5:296. doi: 10.3389/fonc.2015.00296 (PMC4689846; doi:10.3389/fonc.2015.00296)
Supplement: Supplementary file 2 [file table_2.docx]

**Table S2. Quality assessment of selected studies by QUADAS-2.**

| Study | Risk of bias | | | | Applicability concerns | | |
| --- | --- | --- | --- | --- | --- | --- | --- |
|  | Patient selection | Index test | Reference standard | Flow and timing | Patient selection | Index test | Reference standard |
| Bougel 2013 | High | High | Low | High | Low | Low | Low |
| Nikolaidis 2012 | Low | High | Low | Low | Low | Low | Low |
| Eijsink 2012 | High | High | Low | High | Low | Low | Low |
| Eijsink 2011 | High | High | Low | Low | Low | Low | Low |
| Valls-Bautista 2011 | High | High | Unclear | Unclear | Low | Low | Unclear |
| Schache 2010 | High | High | Low | Low | Low | Low | Low |
| Kumari 2009 | High | High | Low | Low | Low | Low | Low |
| Wang 2008 | High | High | Unclear | Unclear | Low | Low | Unclear |
| Pu 2007 | High | High | Low | High | Low | Low | Low |
| Clement 2005 | High | High | Unclear | Unclear | Low | Low | Unclear |

High: high risk, Low: low risk, Unclear: unclear risk
