# Supplementary material for: Diagnostic Value of Methylated Human Telomerase Reverse Transcriptase in Human Cancers: A Meta-Analysis
Source: Front Oncol. 2015 Dec 24;5:296. doi: 10.3389/fonc.2015.00296 (PMC4689846; doi:10.3389/fonc.2015.00296)
Supplement: Supplementary file 3 [file table_3.docx]

**Table S3. Primers and probes for qMSP**

| Author | Amplicon location | Forward Primer (5’-3’) | Probe (5’-3’) | Reverse Primer (5’-3’) | Reference |
| --- | --- | --- | --- | --- | --- |
| Bougel 2013 | -540/-440 | CCC**CGCG**TC**CG**AACCT | **CG**ACCAAAAAAT**CG**C**CG**CA**CG**CA | TT**CG**AGGGAGGGGTTATGATGTG | 20 |
| Eijsink 2012 | -383/-295 | GGTTT**CG**ATAG**CG**TAGTTGTTTC | AAAAAA**CGCG**ACCCAAACCCC**CG**AAT | CTACACCCTAAAAA**CGCG**AAC | 22 |
| Nikolaidis 2012 | -380/-280 | TTGGGAGTT**CG**GTTTGGTTTC | AG**CG**TAGTTGTTT**CG**G | CACCCTAAAAA**CGCG**AA**CG**A | 25 |
| Eijsink 2011 | -383/-295 | GGTTT**CG**ATAG**CG**TAGTTGTTTC | AAAAAA**CGCG**ACCCAAACCCC**CG**AAT | CTACACCCTAAAAA**CGCG**AAC | 23 |
| Schache 2010 | -380/-280 | TTGGGAGTT**CG**GTTTGGTTTC | AG**CG**TAGTTGTTT**CG**G | CACCCTAAAAA**CGCG**AA**CG**A | 27 |
| Pu 2007 | -346/-273 | AAG**CGCG**GTTTAGATTTT**CG** | TT**CG**TT**CG**GAGTAGTTG**CG**TTGT**CG**G | GAATCCACTAAAAACC**CG**ACCTAAC | 26 |

CpG islands were in bold;
