# Supplementary figures and images for: Diagnostic Value of Methylated Human Telomerase Reverse Transcriptase in Human Cancers: A Meta-Analysis
Source: Front Oncol. 2015 Dec 24;5:296. doi: 10.3389/fonc.2015.00296 (PMC4689846; doi:10.3389/fonc.2015.00296)

Figure S1

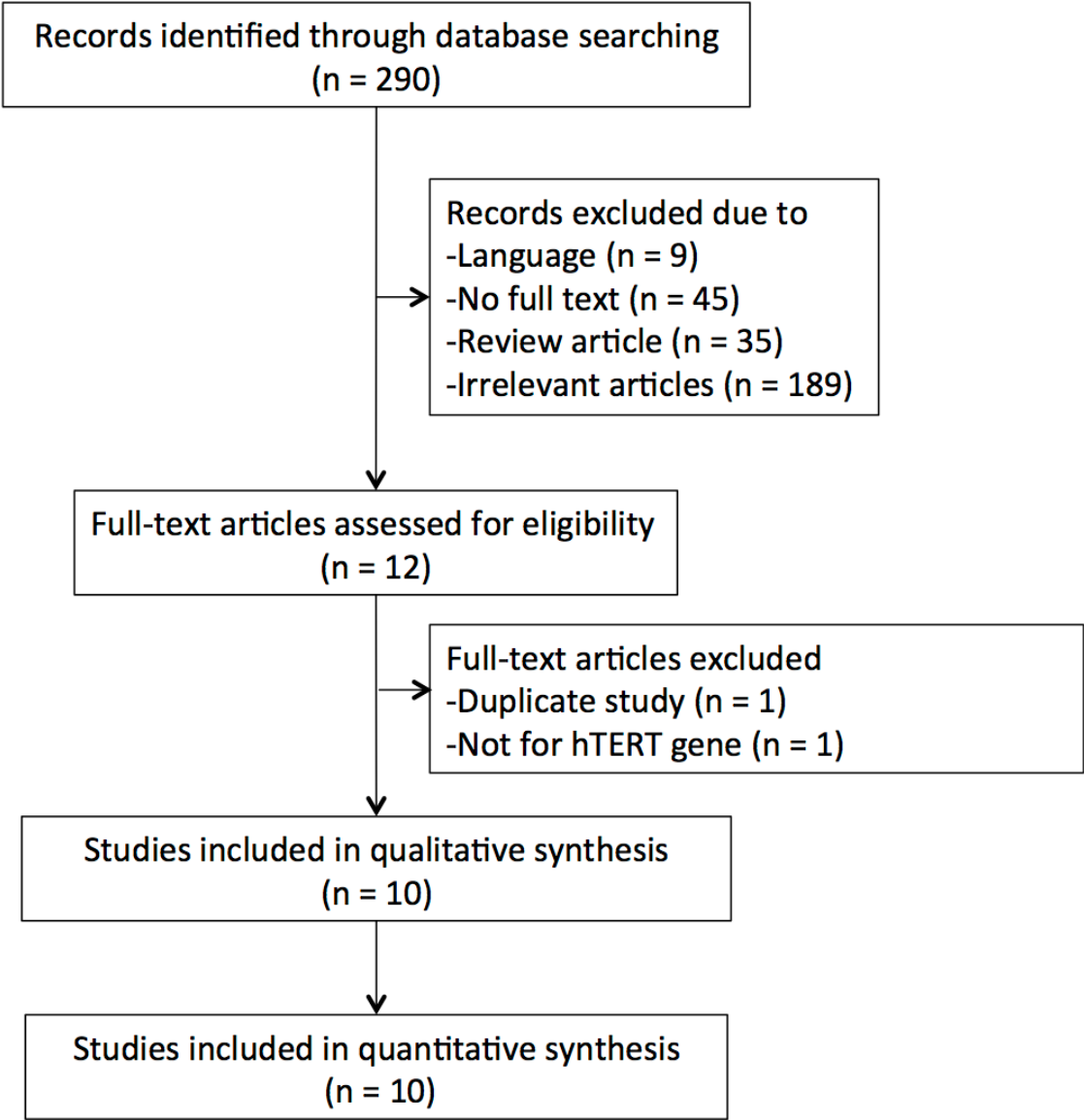

Figure S1 | Flow diagram of study selection.

Supplement: Supplementary file 4 [file image_1.pdf]
