# Supplementary material for: Diagnostic Value of Methylated Human Telomerase Reverse Transcriptase in Human Cancers: A Meta-Analysis
Source: Front Oncol. 2015 Dec 24;5:296. doi: 10.3389/fonc.2015.00296 (PMC4689846; doi:10.3389/fonc.2015.00296)
Supplement: Supplementary file 5 [file image_2.pdf]

Figure S2

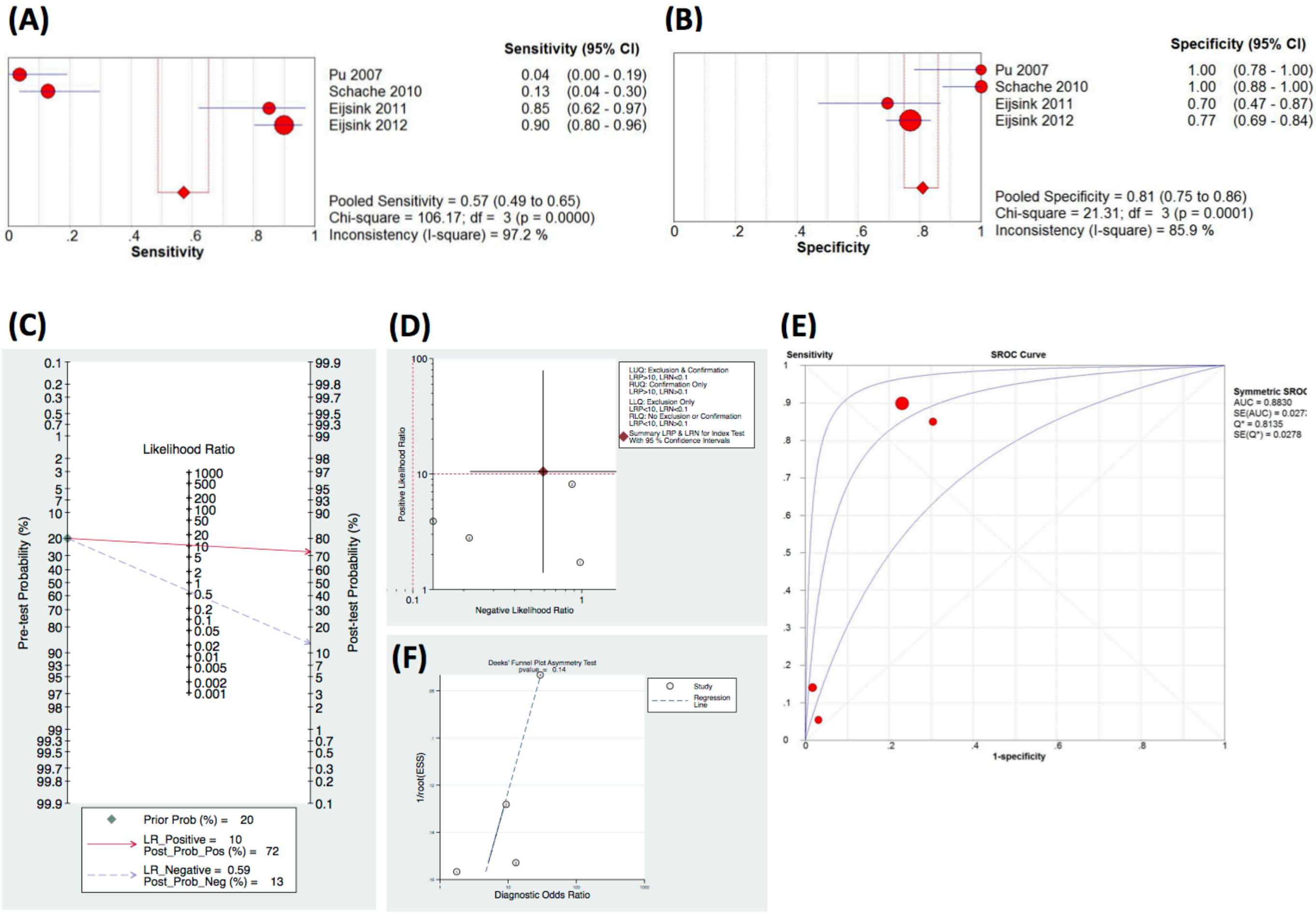

Figure S2 | Diagnostic value of methylated hTERT for discriminating cancer from benign. Forest plot showing the pooled sensitivity (**A**) and specificity (**B**) of methylated hTERT for discriminating cancer from benign. (**C**), Fagan plot displaying post-test probability. (**D**), Likelihood ratio scattergram for confirmation and exclusion. (**E**), SROC curve for diagnostic accuracy. (**F**), Funnel plot with superimposed regression line for testing publication bias.
