# Supplementary material for: Diagnostic Value of Methylated Human Telomerase Reverse Transcriptase in Human Cancers: A Meta-Analysis
Source: Front Oncol. 2015 Dec 24;5:296. doi: 10.3389/fonc.2015.00296 (PMC4689846; doi:10.3389/fonc.2015.00296)
Supplement: Supplementary file 6 [file image_3.pdf]

Figure S3

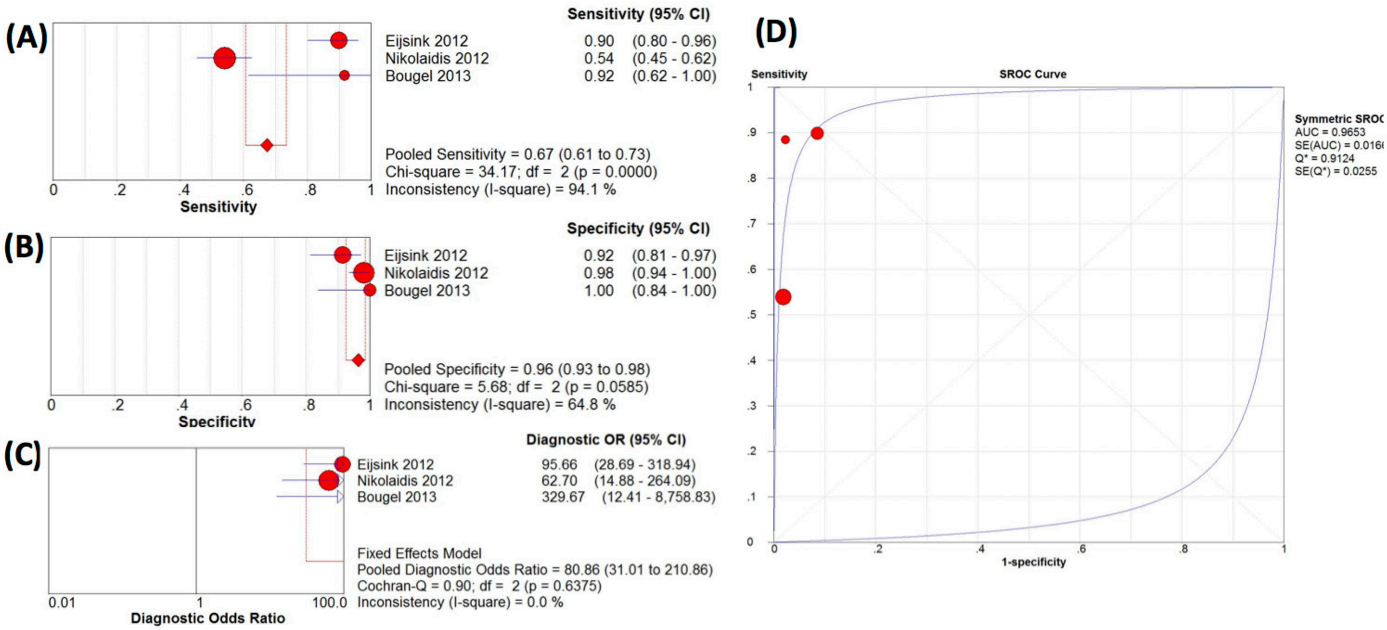

Figure S3 | Diagnostic value of methylated hTERT detected by qMSP for distinguishing cancer from normal. Forest plot of sensitivity (**A**), specificity (**B**) and diagnostic odds ratio (**C**) of methylated hTERT for discriminating cancer from normal. (**D**), SROC curve for diagnostic accuracy.
