# Supplementary material for: Diagnostic Value of Methylated Human Telomerase Reverse Transcriptase in Human Cancers: A Meta-Analysis
Source: Front Oncol. 2015 Dec 24;5:296. doi: 10.3389/fonc.2015.00296 (PMC4689846; doi:10.3389/fonc.2015.00296)
Supplement: Supplementary file 7 [file image_4.pdf]

Figure S4

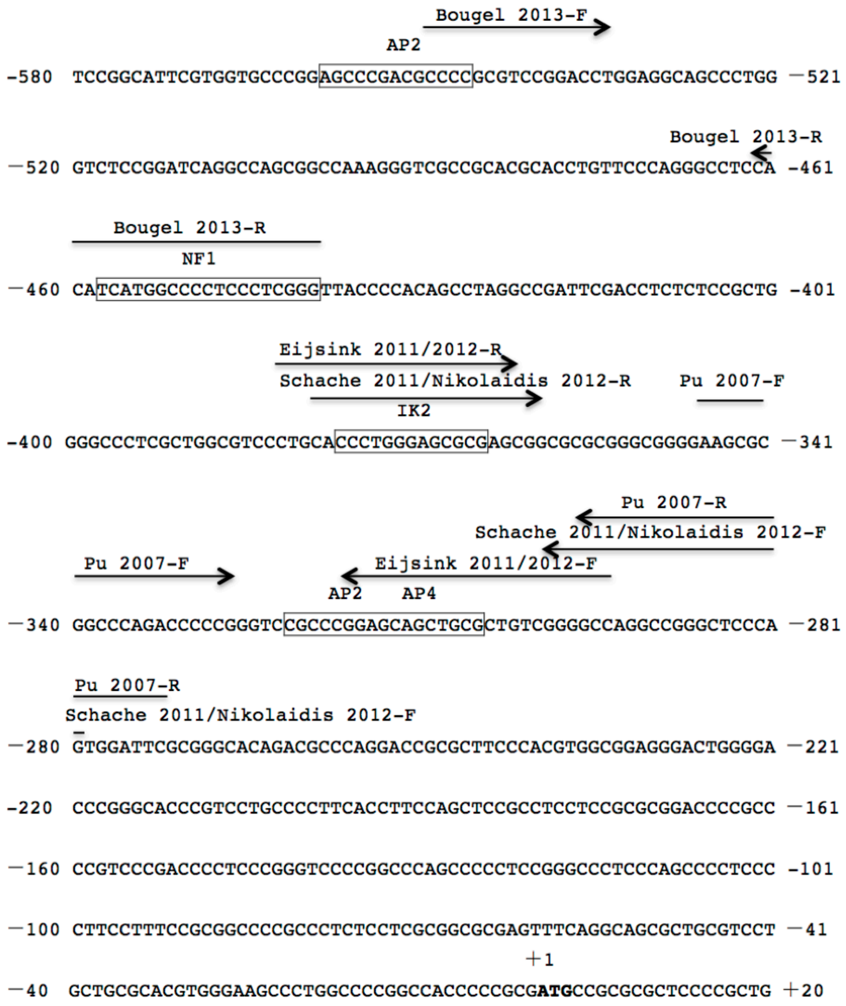

Figure S4 | Nucleotide sequence of hTERT gene regulatory region and the detection sites of different studies using qMSP. The start site for translation is labelled with +1. Putative regulatory sequences were indicated by boxes. The sequences of primers were indicated by arrows.
